# Eruptive Blue Nevi: A Rare Phenomenon Diagnosed With the Aid of Dermoscopy

**DOI:** 10.5826/dpc.1002a41

**Published:** 2020-04-03

**Authors:** Laurent Klapholz, Yuval Ramot

**Affiliations:** 1 Department of Dermatology, Hadassah Medical Center, Hebrew University of Jerusalem, The Faculty of Medicine, Jerusalem, Israel

**Keywords:** blue nevus, melanoma, dermoscopy, dermatology, skin

## Introduction

Sudden change in a pigmented lesion accompanied by the appearance of new lesions is a cause for concern for potential melanoma development. Here we present such a case, diagnosed as eruptive blue nevi in a young woman. This case highlights the importance of dermoscopy for the diagnosis of this rare phenomenon.

## Case Presentation

A 24-year-old woman with no significant medical history presented with a blue lesion on her right shin. The patient reported that the lesion had been present on her leg for many years but had started growing in the preceding 2 months. In addition, she had noticed 2 additional similar, albeit smaller, blue lesions that had appeared on her right thigh and her buttocks 2 weeks before. The lesions were asymptomatic, and the patient denied any additional complaints at the time of examination.

**Figure 1:**
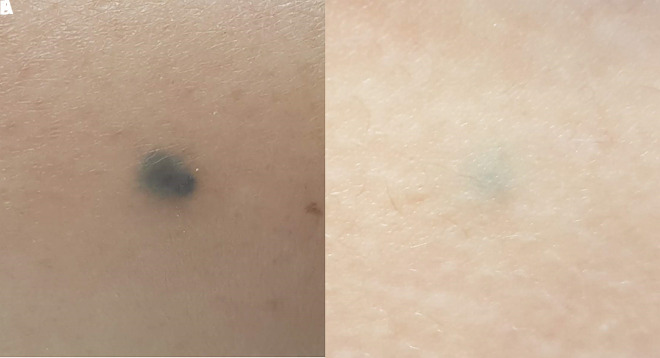
(A) A well-circumscribed, blue-gray macule on the distal lateral aspect of the right shin. (B) A smaller blue lesion on the right thigh.

**Figure 2:**
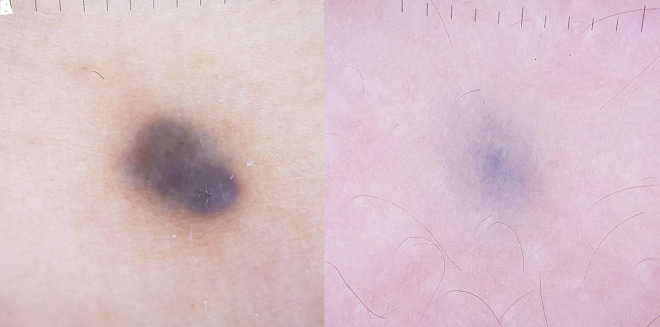
Dermoscopic examination of the lesions on the shin (A) and thigh (B) showing structureless, blue, symmetrical lesions, with a homogenous pattern.

Clinical examination revealed a well-circumscribed, 6 mm in diameter, blue-gray nodule on the distal lateral aspect of the right shin (Figure 1A). Smaller macules, 4 mm in diameter, with a lighter blue color, were present on her right thigh and buttocks (Figure 1B). Dermoscopic examination revealed structureless, blue, symmetrical lesions, with a homogenous pattern (Figure 2). Histopathological examination confirmed the clinical suspicion of a cellular blue nevus, with a Ki-67 proliferation index of less than 1%, and negative HMB45 and MART-1 stainings.

## Conclusions

Blue nevus is a benign neoplasm composed of aberrant melanocytes located in the dermis, which can be found in 0.5%–4% of the white population. It is usually acquired during the second decade of life, presenting as a macule, papule, nodule, or plaque on the buttocks, face, scalp, hands, or feet. Several types of blue nevi exist, including common, cellular, or combined blue nevus; atypical or malignant blue nevus; and large patch/plaque lesions [1].

When a blue nevus develops, it usually remains stable and does not change with time. Furthermore, the presence of multiple blue nevi is a rare phenomenon and usually presents as aggregated or plaque-type lesions. So far, there have been only 13 reports of eruptive blue nevi, where the number of nevi varied from 3 to hundreds [2]. In general, but also in the case of eruptive blue nevi, the sudden eruption of multiple nevi is observed following physiological conditions such as pregnancy or pathological disorders such as sunburn or trauma.

Although the clinical and dermoscopic features of the lesions in the patient described here were typical for a blue nevus, the sudden change and the appearance of new lesions in a short period of time were suspicious, especially when considering the importance of the blue hue as part of the dermoscopic features of malignancy. This, together with the lack of any triggering factor, warranted histopathological examination of the lesion to rule out malignancy.

Here we presented a rare case of eruptive blue nevi in a patient with no known triggering factors. It highlights the need for careful inspection of these lesions to rule out melanoma or malignant blue nevus, especially since melanoma has been reported to mimic benign blue nevus.
